# Study on Mechanical Properties and Synergistic Mechanism of Concrete Reinforced with Hybrid Basalt Fibers of Different Lengths

**DOI:** 10.3390/ma19132848

**Published:** 2026-07-03

**Authors:** Yingying Tao, Chuan Zhao, Yanmei Zhang, Yanchang Zhu, Yongxiang Fang, Rui Zhang, Qikai Wang, Fuxing Wu, Qingzhe Yi

**Affiliations:** 1 College of Pipeline and Civil Engineering, China University of Petroleum (East China), Qingdao 266580, China; taoyingyingupc@163.com (Y.T.); yanchangzhu2025@163.com (Y.Z.); xuechunyang61@gmail.com (Y.F.); rui277940@163.com (R.Z.); 13153398315@163.com (Q.W.); 18354930651@163.com (F.W.); 15169820377@163.com (Q.Y.); 2 Sichuan Academy of Water Conservancy, Chengdu 610072, China; zhaochuanvip@163.com

**Keywords:** basalt fiber-reinforced concrete, hybrid fibers, mechanical properties, synergistic mechanism, numerical simulation

## Abstract

Basalt fiber (BF) is an effective reinforcement for improving concrete’s mechanical properties and crack resistance due to its high tensile strength and bridging ability. To investigate the influence of fiber length combinations on the mechanical performance of concrete, basalt fiber-reinforced concrete (BFRC) specimens were prepared using single and hybrid blending methods. Compressive and splitting tensile tests, scanning electron microscopy, and numerical simulations were conducted to evaluate the effects of fiber content and length hybridization, and analyze the possible reinforcement mechanisms. Results showed that for single-blended BFRC with 18 mm BF, both compressive and tensile strengths peaked at a 0.2% dosage, then declined. Conversely, the strengths of hybrid BFRC continuously increased with fiber content, reaching 33.00 MPa and 2.38 MPa at a 0.3% dosage, significantly outperforming the single-length fiber systems. Microstructural observations and numerical analyses suggested that fibers with different lengths contributed to complementary reinforcement effects during the loading process. The improved performance was attributed to the combined effects of crack bridging and stress redistribution provided by fibers with different lengths.

## 1. Introduction

Concrete, as a core structural material, plays a dominant role in engineering applications such as bridges, tunnels, and dams due to its high compressive strength, favorable durability, and adaptability to complex working conditions [1]. However, the quasi-brittle nature of conventional concrete makes it susceptible to micro-cracks under loading, leading to insufficient crack resistance and toughness, which restricts its application in high-performance engineering [2,3]. Therefore, improving the mechanical properties, crack resistance, and toughness of concrete has become an urgent problem to be addressed in the engineering field.

To mitigate the brittle failure characteristics of concrete, fiber-reinforced concrete incorporates fibers to provide distributed micro-reinforcement, which can restrain the initiation and propagation of micro-cracks, thereby significantly enhancing compressive strength, tensile strength, and ductility. Acting as “micro-rebars” within the concrete, fibers improve internal stress distribution and delay crack development through bridging effects and stress transfer [4]. Compared with steel and polypropylene fibers, basalt fiber (BF) possesses advantages such as high strength, high elastic modulus, corrosion resistance, and eco-friendliness, making it a promising new reinforcement material. Existing studies have demonstrated that incorporating an appropriate amount of BF can significantly enhance the compressive strength, tensile strength, and crack resistance of concrete [5]. Wu et al. [6] found that the addition of BF effectively enhanced the toughness and crack resistance of concrete, increasing the compressive strength by up to 4.48%. Zeng et al. [7] pointed out that BF could significantly reduce the porosity and the proportion of harmful pores in concrete, thereby improving its durability and compressive strength. Fu et al. [8] reported that BF could markedly improve the tensile strength and compressive toughness of concrete, contributing to the reinforcement of the crack-arresting mechanism. However, fibers of a single length have limitations in controlling cracks. Short fibers are beneficial for suppressing micro-crack initiation but exhibit limited bridging capacity; conversely, long fibers can span macro-scale cracks but may lead to poor dispersion and fiber entanglement [9,10,11,12]. Although hybrid BFs with different lengths have been investigated in previous studies [13,14], the interaction between fiber length combinations and their effects on crack evolution, mechanical enhancement, and meso-scale stress transfer mechanisms remain insufficiently clarified.

Therefore, based on the baseline concrete mix proportion provided by a dike project in Sichuan, this study investigates the effects of fiber incorporation types (single and hybrid), dosages, and lengths (for single blending) on the mechanical properties and internal stress evolution of basalt fiber-reinforced concrete (BFRC). This is achieved through compressive tests, splitting tensile tests, scanning electron microscopy (SEM) observations, and numerical simulations. The influence mechanisms of hybrid BFs on concrete performance are revealed from multiple perspectives, including macro-mechanical properties, microstructural characteristics, and meso-stress evolution, providing a reference for the engineering application of hybrid BFRC in hydraulic concrete structures.

## 2. Materials and Methods

### 2.1. Materials and Mix Proportions

The concrete reference mix proportion used in this paper was formulated by a construction unit of a dike project in Sichuan Province with reference to the “Specification for Mix Proportion Design of Ordinary Concrete” (JGJ 55-2011) [15]. The amounts of water, cement, sand, crushed stone and water-reducing agent were 182, 330, 964, 964 and 3 kg·m^−3^, respectively. P.O 42.5 ordinary Portland cement was used as cement, limestone crushed stone was used as coarse aggregate, and natural river sand was used as fine aggregate. The fineness modulus was 2.9 as measured by sieve analysis. The chemical admixture was a retarding polycarboxylate superplasticizer. The mixing water was municipal tap water.

**Figure 1:**
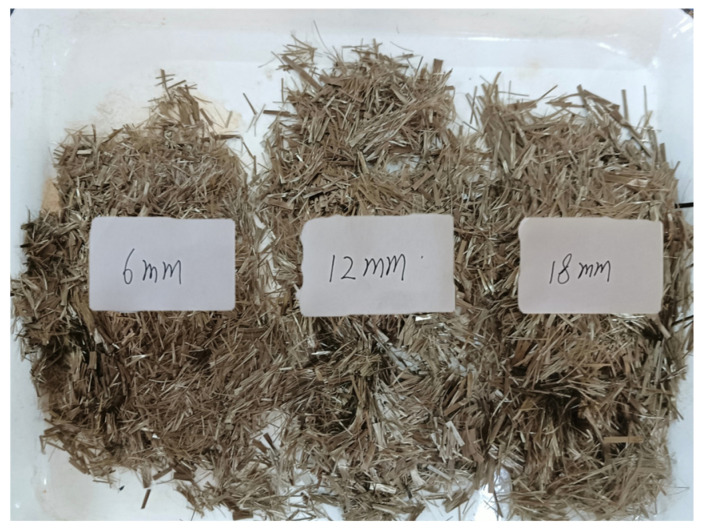
Basalt fibers of different lengths.

Referring to the relevant literature [16] and combined with the results of multiple trial mixes at the construction site, three lengths of BF, 6 mm, 12 mm and 18 mm, as shown in Figure 1, were selected. The BFs have a monofilament diameter of 20 μm, density of 2800 kg·m^−3^, tensile strength of 2500 MPa, elastic modulus of 76 GPa, and elongation at break of 3.0%. Four levels of fiber content were set: 0%, 0.1%, 0.2%, and 0.3%. The groups with no fiber content (X0 group), single-length fiber group (Group XV), and hybrid fiber group (Group XW) were designed. Among them, the Group XV contained only 18 mm BF, while the Group XW consisted of hybrid BFs with lengths of 6 mm, 12 mm, and 18 mm. The hybrid fibers were incorporated based on a mass ratio of 3:4:3 (BF-6 mm:BF-12 mm:BF-18 mm), which was selected with reference to previous studies [17]. For each total fiber content level (0.1%, 0.2%, and 0.3%), the corresponding amounts of each fiber length were calculated according to this ratio, as summarized in Table 1.

### 2.2. Specimen Preparation

**Figure 2:**
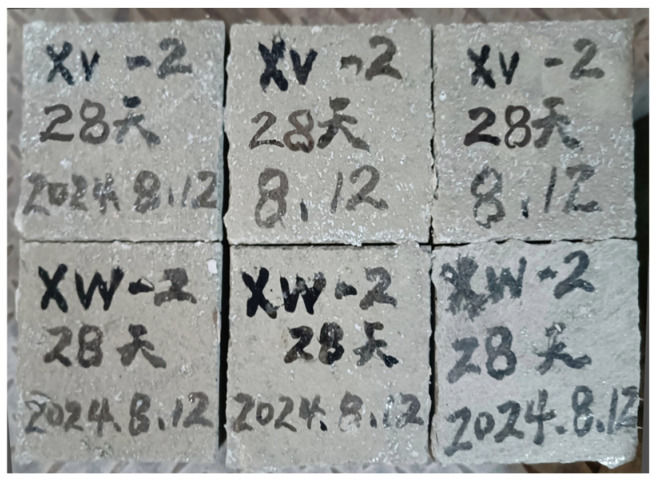
Partially reinforced BFRC. The Chinese characters “28天” on the specimen indicate a curing age of 28 days.

The specimens for the compressive strength test and splitting tensile test were all made of standard cubic size of 150 × 150 × 150 mm. The specimen molding and curing system strictly followed the “Test Procedure for Hydraulic Concrete” (SL/T 352-2020) [18], and the process was as follows: The specimens were prepared by graded feeding; in the dry mixing stage, the aggregate and cementitious material were mixed evenly first, and then BF was added in batches to avoid agglomeration; then the water-reducing agent solution and mixing water were added and stirred for 90 s. After mixing, the specimens were poured into a 150 mm cubic mold, and after vibration molding, they were placed under standard curing conditions (temperature 20 ± 2 °C, humidity ≥ 95%) for 24 h, and after demolding, they were cured for 28 days. Some BFRC molded specimens are shown in Figure 2. Three specimens were prepared for each mixture proportion, and the average value was taken as the representative result.

### 2.3. Test Methods

Uniaxial compressive strength and splitting tensile strength tests were conducted using a YAW-2000 microcomputer servo compression testing machine (Shandong Maijie Testing Equipment Co., Ltd., Jinan, China), equipped with a 2000 kN load cell. The compressive strength test was performed according to ISO 1920-3:2019, and a constant loading rate of 0.5 MPa/s was applied until specimen failure. The splitting tensile strength test was conducted according to SL/T 352-2020 [19], with a constant loading rate of 0.05 MPa/s. The peak load and failure mode were recorded for each specimen.

**Figure 3:**
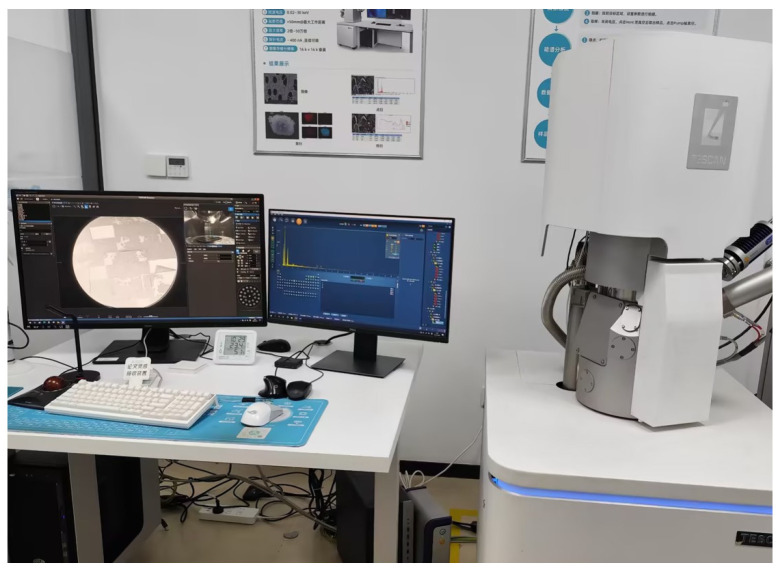
Scanning electron microscope.

To further analyze the microstructural characteristics of BFRC and the fiber–matrix interface mechanism, microscopic morphology observation of the specimen cross-section was performed using a MIRA LMS scanning electron microscope (TESCAN, Brno, Czech Republic), as shown in Figure 3. Samples from typical failure areas were selected, dried, and fixed onto a metal sample stage. Gold sputtering was then performed using a QuorumSC7620 sputtering coating system (Quorum Technologies Ltd., Lewes, UK) to improve sample conductivity and reduce the impact of charge effects on imaging quality. During SEM testing, the accelerating voltage was set to 3 kV to reduce thermal damage to hydration products and micro-crack structures from the electron beam.

## 3. Results and Discussion

### 3.1. Effect of Fiber Incorporation Type on Compressive Strength

**Figure 4:**
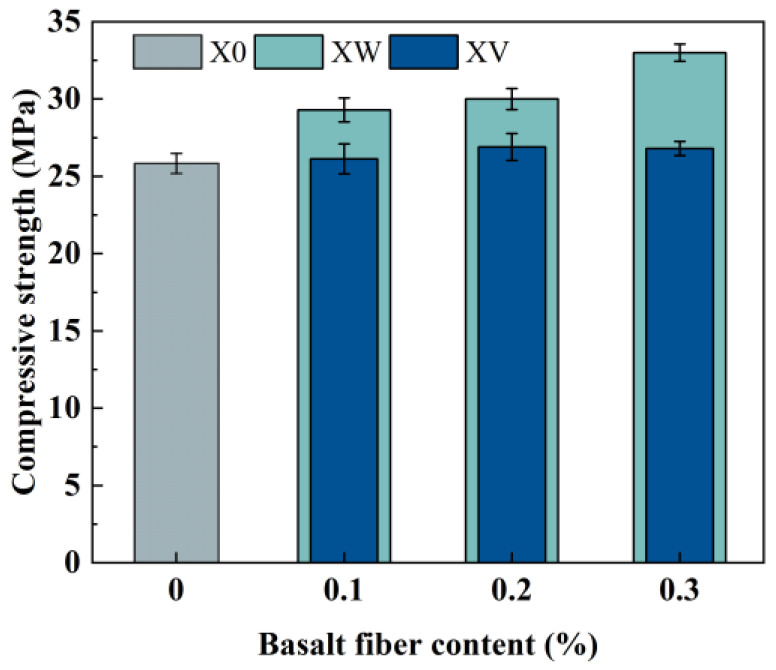
Compressive strength of BFRC specimens (error bars represent standard deviation).

Figure 4 displays the compressive strength test results of BFRC. The error bars represent the standard deviation obtained from three parallel specimens. The corresponding mean values, standard deviations (SD), and coefficients of variation ($C_{v}$) are summarized in Table 2.

The compressive strength of Group XV exhibited an initial increase followed by a decrease with increasing fiber dosage. The compressive strength in this single-length fiber system reached its peak value of 26.9 MPa at a fiber dosage of 0.2%, representing an increase of approximately 4.1% compared with the plain concrete Group X0. The 18 mm BFs have a relatively high aspect ratio, which enables them to provide stronger crack-bridging effects within the concrete matrix. This reinforcement effect improves crack resistance and stress transfer capacity, thereby enhancing the compressive strength of concrete. The compressive strength of Group XV stopped growing and even showed a slight decline when the fiber dosage increased further to 0.3%. This reduction may be attributed to the increased possibility of fiber entanglement and local agglomeration at higher fiber contents, which affects fiber dispersion and introduces local weak regions inside the matrix.

Group XW exhibited a different trend in compressive strength compared with Group XV. The compressive strength continuously increased with increasing fiber content and reached 33.00 MPa at 0.3% fiber dosage. Compared with Group XV, the hybrid fiber system showed improved reinforcement efficiency, with the compressive strength increased by 23.1% compared with X0 and 23.1% compared with XV3.

Several possible factors may contribute to the strength improvement of the hybrid BFRC. A reasonable length combination may improve fiber utilization efficiency by reducing the limitation of single-length fibers [20,21]. Furthermore, this enhanced performance may be attributed to the complementary roles of fibers with different lengths during crack development. Short fibers are more effective in restraining micro-crack growth, while longer fibers provide crack-bridging capacity for larger cracks [22]. These effects contribute to the continuous increase in compressive strength of the hybrid fiber system with increasing fiber dosage, resulting in better reinforcement performance compared with the single-length fiber system.

Statistical analysis further indicates that the coefficient of variation of the compressive strength results ranged from 1.71% to 3.72% for the XV groups and from 1.69% to 2.64% for the XW groups, suggesting acceptable experimental repeatability. Furthermore, one-way analysis of variance (ANOVA) was performed to evaluate the statistical differences among the mixture groups. The results showed that the compressive strength varied significantly among the tested groups (F = 38.428, *p* < 0.001). The relatively low variation values and ANOVA results indicate that the differences among groups were statistically significant and mainly associated with fiber incorporation.

### 3.2. Effect of Fiber Incorporation Type on Splitting Tensile Strength

**Figure 5:**
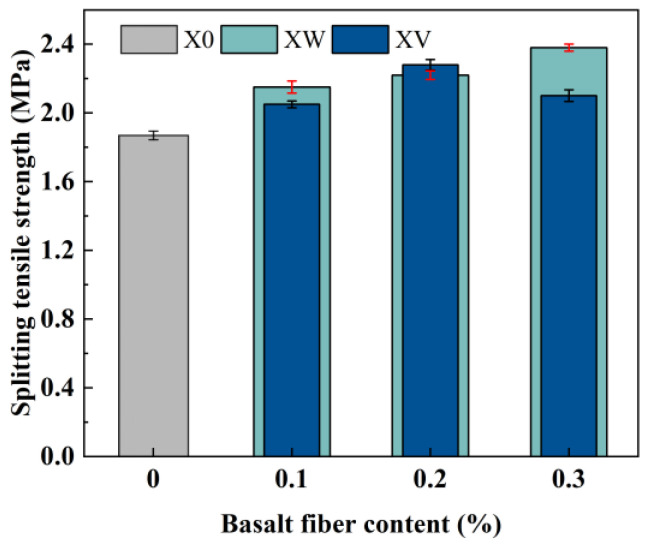
Splitting tensile strength of BFRC specimens (error bars represent standard deviation).

Figure 5 illustrates the splitting tensile strength of BFRC. The error bars represent the standard deviation obtained from three parallel specimens. The statistical results including mean values, standard deviations (SD), and coefficients of variation ($C_{v}$) are listed in Table 3.

Overall, the addition of BF improved the splitting tensile strength of concrete. Group XV showed an initial increase followed by a decrease with increasing fiber dosage. The maximum splitting tensile strength was obtained at 0.2% fiber content, reaching 2.06 MPa, which was approximately 10.8% higher than that of the plain concrete group.

At higher fiber content, the reduction in tensile strength may be associated with the increased interaction between fibers and the reduced uniformity of fiber distribution. Excessive fiber addition can decrease the effectiveness of crack bridging by increasing the probability of fiber clustering.

Compared with Group XV, the hybrid fiber system showed a more stable enhancement trend. The splitting tensile strength of Group XW increased continuously with increasing fiber content and reached 2.23 MPa at 0.3% fiber content. This value was approximately 19.9% higher than that of plain concrete and 17.4% higher than XV3.

The coefficient of variation of splitting tensile strength ranged from 1.07% to 1.79% for Group XV and from 0.94% to 1.77% for Group XW, demonstrating good consistency among repeated tests. One-way ANOVA also showed significant differences in splitting tensile strength among the tested groups (F = 67.738, *p* < 0.001), indicating that the changes in tensile performance were related to fiber incorporation rather than experimental variability.

### 3.3. Microstructure Analysis

#### 3.3.1. Influence of Basalt Fibers on Matrix Morphology

**Figure 6:**
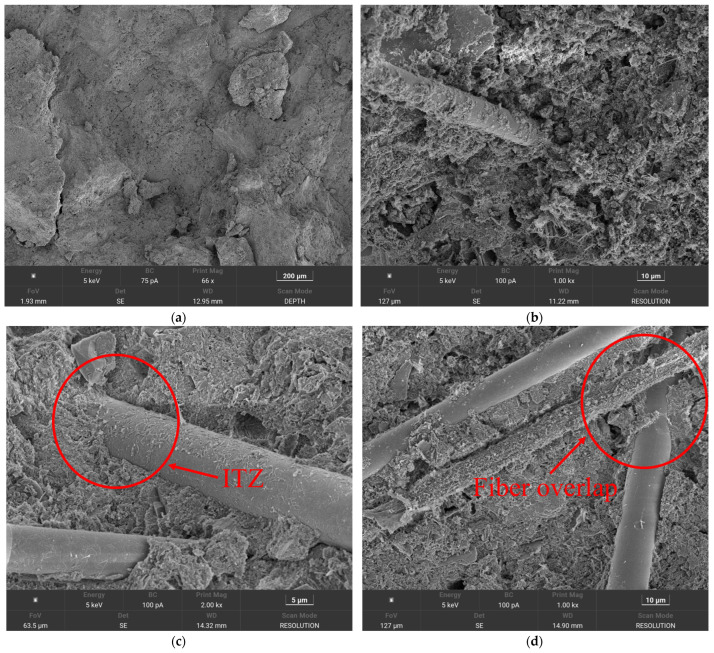
SEM images of plain concrete and single-blended BFRC. (**a**) X0 (66×, scale bar: 200 μm); (**b**) XV1 (1000×, scale bar: 10 μm); (**c**) XV2 (2000×, scale bar: 5 μm); (**d**) XV3 (1000×, scale bar: 10 μm).

**Figure 7:**
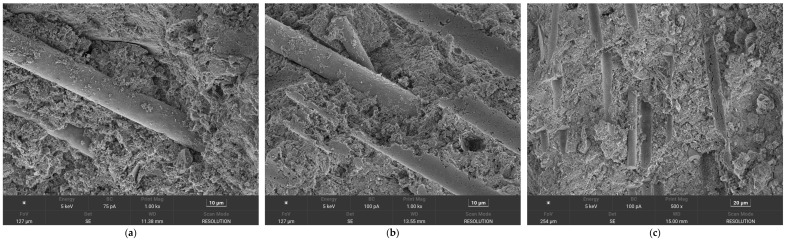
SEM images of hybrid BFRC. (**a**) XW1 (1000×, scale bar: 10 μm); (**b**) XW2 (1000×, scale bar: 10 μm); (**c**) XW3 (500×, scale bar: 20 μm).

SEM observations were used to qualitatively evaluate the influence of BF on the microstructural characteristics of BFRC. Figure 6 and Figure 7 show the micro-morphological features of plain concrete, single-length BFRC, and hybrid BFRC.

At the observed scale, plain concrete exhibited more visible pores and micro-cracks, indicating a relatively loose microstructural morphology (Figure 6a). At a fiber dosage of 0.1%, fewer fibers were observed within the matrix, and limited contact between fibers and surrounding hydration products was observed (Figure 6b). When the dosage increased to 0.2% (Figure 6c), more fibers were observed in the matrix, and hydration products were attached to the fiber surfaces, suggesting improved contact between fibers and the surrounding cementitious matrix. At a dosage of 0.3% (Figure 6d), local fiber agglomeration and overlapping were observed, which may reduce the effectiveness of fiber reinforcement by affecting fiber dispersion and creating potential weak regions in the matrix.

Compared with the single-length fiber group, hybrid BFRC showed a more complex fiber distribution with different fiber lengths within the observed regions (Figure 7). The combination of fibers with different lengths may contribute to improved reinforcement efficiency by providing different levels of crack resistance. According to previous studies, shorter fibers are generally more effective in restricting the initiation and development of micro-cracks, while longer fibers can provide bridging effects after crack formation. These complementary contributions may partly explain the improved mechanical performance of hybrid BFRC.

Although SEM observations provide qualitative evidence of fiber–matrix interactions and microstructural changes, the interfacial transition zone (ITZ) thickness, chemical composition, and fiber distribution characteristics were not quantitatively characterized in this study. Therefore, the SEM results are considered as supplementary qualitative evidence to support the observed mechanical performance trends rather than direct quantitative verification of the reinforcement mechanism.

#### 3.3.2. Quantitative Analysis of Pore Structure

SEM images were analyzed using ImageJ 1.54g software to evaluate the pore distribution characteristics of BFRC. The SEM images were first converted into grayscale images and then binarized to distinguish pore regions from the solid matrix. The scale calibration was performed based on the scale bar information of each SEM image. The Analyze Particles function was then used to identify pore regions and calculate their area fractions.

To improve the reliability of the analysis, representative regions were selected from each mixture group according to the following criteria: (1) avoiding obvious fiber agglomeration areas; (2) excluding specimen edges and damaged regions; and (3) maintaining comparable observation conditions. SEM images with a magnification of 1000× were selected for pore analysis. Representative SEM regions were analyzed for each mixture group, and the total analyzed area was approximately 1000–2000 μm^2^ depending on the selected regions.

The pore ratio obtained from SEM image processing was defined as two-dimensional apparent porosity *P_a_*, which represents the pore area fraction within the analyzed section:

(1) $$P_{a}=\frac{\sum A_{i}}{A_{t}}$$

where $\sum A_{i}$ is the total identified pore area and $A_{t}$ is the total analyzed area.

**Figure 8:**
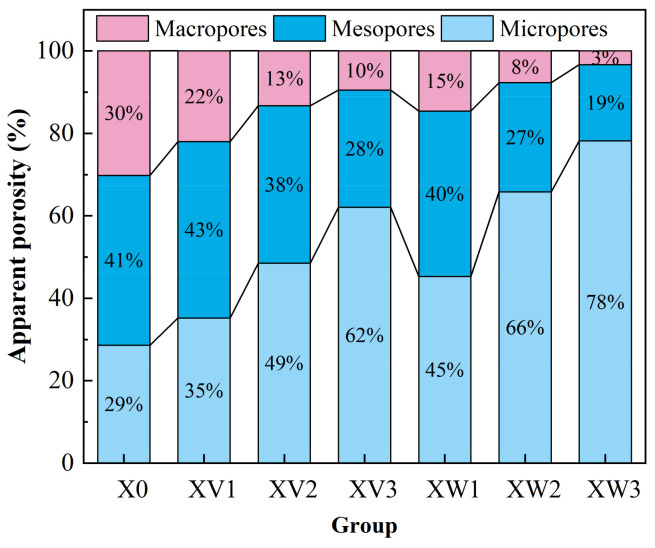
Pore proportions of BFRC under different fiber contents.

According to the equivalent pore diameter, pores were classified into three categories: micropores (<10 μm), mesopores (10–50 μm), and macropores (>50 μm). Figure 8 presents the pore size distribution characteristics of BFRC with different fiber contents.

The image analysis results indicate that BF addition affected the pore distribution characteristics of concrete. Compared with the plain concrete group (X0), BFRC groups exhibited a lower proportion of macropores and an increased proportion of micropores. For the single-length fiber group (XV), the micropore proportion increased from 35.2% to 62.1% with increasing fiber content, while the macropore proportion decreased from 22.0% to 9.5%. Meanwhile, the apparent porosity decreased from 8.45% to 4.28%.

For the hybrid fiber group (XW), the micropore proportion further increased with fiber dosage, reaching 78.2% at 0.3% fiber content, while the macropore proportion decreased to 3.3%. The apparent porosity decreased to 2.85%. These results indicate that fiber incorporation may contribute to reducing the proportion of larger pores and modifying the pore size distribution. However, since SEM-based analysis reflects only local two-dimensional characteristics, further three-dimensional characterization is required to fully describe the pore structure evolution.

## 4. Numerical Simulation

A three-dimensional finite element analysis method served to model the materials. ABAQUS CAE 2022 software helped construct the meso-scale model of BF and the concrete matrix. A Python 3.8.10 script generated the random distribution of the fibers. This script combined with the concrete constitutive model to describe the mechanical interactions between the fibers and the matrix.

### 4.1. Model Establishment

The concrete matrix was simulated using the concrete damaged plasticity (CDP) model available in ABAQUS [23,24]. The CDP model is a continuum damage-based constitutive model that can effectively describe the nonlinear mechanical behavior of concrete under tensile cracking and compressive crushing. In this model, the plastic deformation and stiffness degradation caused by irreversible damage are considered simultaneously.

The stress–strain relationship of concrete in the CDP model can be expressed as:

(2) $$\sigma =(1-d)E_{0}(\varepsilon -\varepsilon^{pl})$$

where σ is the stress; $E_{0}$ is the initial elastic modulus of concrete; ε and $\varepsilon^{pl}$ are the total strain and plastic strain, respectively; d represents the damage variable, ranging from 0 (undamaged state) to 1 (complete failure).

The tensile and compressive damage variables were introduced to describe the stiffness degradation during loading. The tensile damage variable $d_{t}$ represents the degradation caused by crack initiation and propagation, while the compressive damage variable $d_{c}$ represents the degradation associated with concrete crushing. Their evolution can be described as:

(3) $$d_{t}=1-\frac{\sigma_{t}}{\sigma_{tu}}$$

(4) $$d_{c}=1-\frac{\sigma_{c}}{\sigma_{cu}}$$

where $\sigma_{t}$ and $\sigma_{c}$ are the current tensile and compressive stresses, respectively; $\sigma_{tu}$ and $\sigma_{cu}$ are the corresponding peak strengths.

The concrete tensile and compressive constitutive relationships were defined according to the experimental mechanical response of BFRC and the CDP model requirements. The CDP parameters adopted in the simulation are listed in Table 4, including the dilation angle, eccentricity, $f_{b0}/f_{c0}$, K, and viscosity parameter.

The dilation angle controls the expansion behavior of concrete under plastic deformation. The eccentricity determines the shape of the plastic potential surface, while the ratio $f_{b0}/f_{c0}$ and parameter K control the yield surface evolution. The viscosity parameter was introduced to improve numerical convergence during the nonlinear analysis.

BF mainly contributes to tensile resistance and crack bridging during the loading process. Since BF exhibits approximately linear elastic behavior before fracture, a linear elastic constitutive model was adopted for BF [25]. The stress–strain relationship of BF was defined as:

(5) $$\sigma_{f}=E_{f}\varepsilon_{f}$$

where $\sigma_{f}$ is the fiber stress, $E_{f}$ is the elastic modulus of BF, and $\varepsilon_{f}$ is the fiber strain.

The relevant material parameters used in the numerical simulation are summarized in Table 4.

BF possessed a uniform cylindrical structure. The fiber diameter was 0.02 mm. The length ranged between 6 mm and 21 mm. A Python script generated the random distribution model of the fibers. The input parameters included the specimen size, fiber quantity, fiber length, and fiber diameter. These parameters achieved the random spatial distribution of the fibers inside the concrete matrix. The generation process of the random fiber distribution included the following steps [26]:

(1) Fiber position generation: The simulation zone was a cube with a side length of *L*. The script randomly generated the starting coordinates ($x_{s},y_{s},z_{s}$) of the fibers inside this zone. The coordinates satisfied a uniform distribution:
  (6) $$x_{s},y_{s},z_{s}\sim \mathrm{Uniform}(0,L)$$
(2) Fiber orientation generation: The angles θ and ϕ in a spherical coordinate system defined the fiber orientation. The angle θ was the angle with the *x*-axis. The angle ϕ was the angle with the *z*-axis, as illustrated below:
  (7) θ~Uniform(0,2π), ϕ~Uniform(0,π)

The fiber endpoint coordinates ($x_{e},y_{e},z_{e}$) are determined based on the fiber length $L_{f}$ and its orientation angles:

(8) $$\left\{\begin{array}{l} x_{e}=x_{s}+L_{f}\sin\phi \cos\theta \\ y_{e}=y_{s}+L_{f}\sin\phi \sin\theta \\ z_{e}=z_{s}+L_{f}\cos\phi \end{array}\right.$$

(3) Overlap inspection: Fibers and coarse aggregates must avoid overlap. The inspection checked the distance between the fibers and the aggregates. The minimum distance D from the fiber endpoints or midpoints to the aggregate center required a specific calculation. The calculation ensured the following relationship:
  (9) $$D\geq R_{a}$$
  where $R_{a}$ is the aggregate radius. If a fiber overlaps with an aggregate, its initial position and orientation are regenerated until the overlap is eliminated. For polyhedral aggregates, a geometric algorithm is used to check whether the fiber intersects any aggregate face, ensuring the fiber is fully embedded within the matrix.
(4) Fiber volume fraction control: The fiber volume fraction $V_{f}$ is regulated by adjusting the number of fibers $N_{f}$, calculated as follows:
  (10) $$V_{f}=\frac{N_{f}\cdot \pi {\left(\frac{D_{f}}{2}\right)}^{2}\cdot L_{f}}{L^{3}}$$

**Figure 9:**
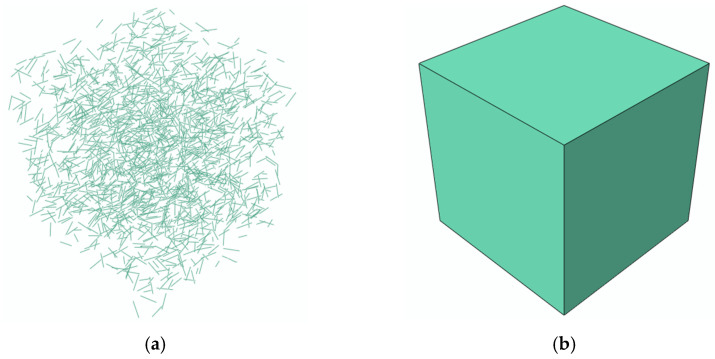
BFRC numerical analysis model. (**a**) BF; (**b**) concrete.

The model generated the spatial positions and orientations of the fibers randomly. This randomness guaranteed a reasonable fiber distribution. An overlap detection algorithm ran at the same time. This algorithm prevented interception or overlapping among the fibers. The control of the fiber quantity determined the fiber volume fraction. Different fiber contents corresponded to different numbers of random fiber elements. Figure 9 shows the random BF distribution model and the concrete matrix established in ABAQUS.

**Figure 10:**
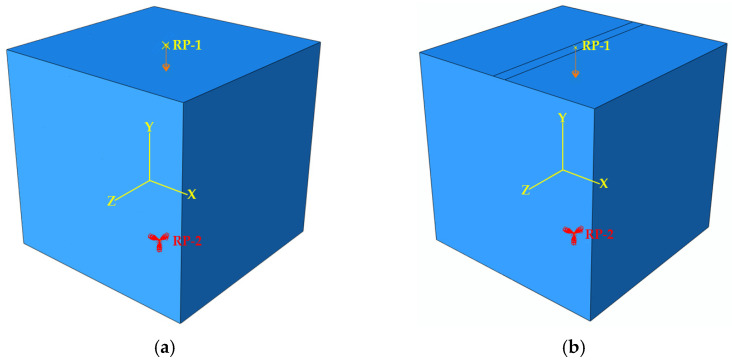
Boundary conditions of the BFRC numerical model. (**a**) Compression; (**b**) Splitting tension.

The compression simulation used a static general analysis step. This step simulated the mechanical response of BFRC under compressive loads. The geometric nonlinearity option was turned on. The splitting tensile simulation utilized a dynamic implicit analysis step. This specific step improved the calculation stability during the crack propagation stage. The simulation used a 150 mm cube specimen. The loading direction was along the *y*-axis (vertical direction). The compression model achieved displacement loading through the coupling between the upper and lower surface reference points and the loading surfaces. The splitting tensile model set a loading zone with a width of 10 mm on the upper and lower surfaces of the specimen along the *x*-axis direction. This model established reference points for coupling constraints. This setup simulated the bearing strip loading method. The bottom reference point possessed a completely fixed constraint. The top reference point received a displacement load along the negative *y*-axis direction. Figure 10 shows the boundary condition settings.

**Figure 11:**
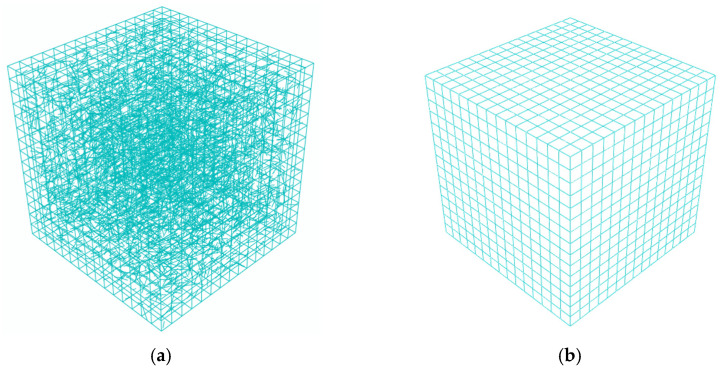
Mesh generation of the BFRC numerical model. (**a**) BFRC; (**b**) plain concrete.

The mesh generation of the concrete matrix used C3D8R eight-node reduced integration elements. The mesh size of the concrete matrix was 10 mm. The BF utilized T3D2 truss elements. The mesh size of the BF was 3 mm. Fibers were generated randomly as independent parts. An embedded region constraint embedded the fibers into the concrete matrix. This constraint considered the reinforcement effect of the fibers on local mechanical properties. Figure 11 shows the mesh generation of the BFRC and plain concrete models.

### 4.2. Model Validation and Solution

**Figure 12:**
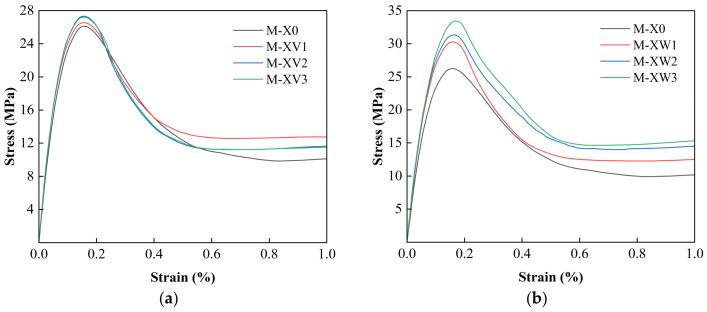

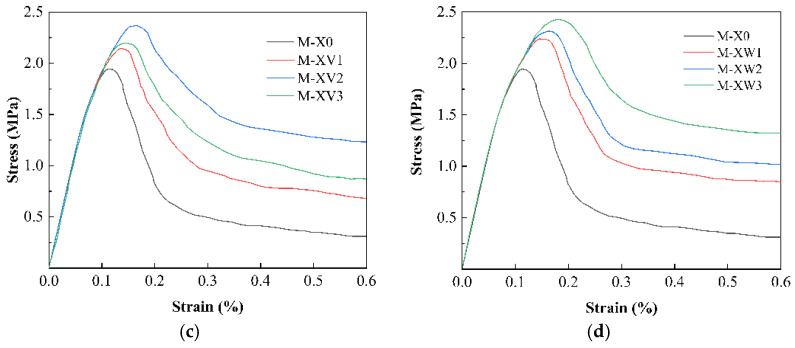
Stress–strain curves of BFRC under different fiber contents. (**a**) Compression-single fiber; (**b**) compression-hybrid; (**c**) splitting tension-single fiber; (**d**) splitting tension-hybrid.

**Figure 13:**
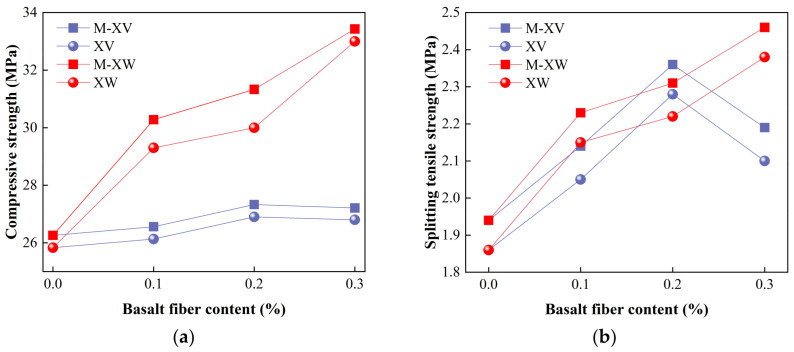
Experimental and simulated mechanical strength results of BFRC. (**a**) Compression; (**b**) splitting tension.

The numerical simulation results and experimental results of BFRC under compression and splitting tension are compared in Figure 12 and Figure 13. The M series represents the numerical simulation results. Figure 12 shows the compressive and splitting tensile stress–strain curves of plain concrete, single-length fiber concrete, and hybrid fiber concrete.

Compared with plain concrete, BFRC exhibited higher peak stress and peak strain, indicating that BF improved the load-bearing capacity and deformation capacity of concrete. The post-peak stress degradation became slower, which suggests that the incorporation of fibers can delay crack development and improve the post-peak response of concrete. The hybrid fiber group showed improved mechanical behavior compared with the single-length fiber group, indicating that different fiber configurations influence the reinforcement performance of BFRC.

Figure 13 presents the comparison between the simulated and experimental mechanical strength results. The numerical model captured the variation trends of BFRC mechanical properties under different fiber contents and configurations. The simulated results showed consistent trends with the experimental results for both compressive strength and splitting tensile strength.

To further evaluate the accuracy of the numerical model, the mean absolute error (*MAE*), root mean square error (*RMSE*), mean absolute percentage error (*MAPE*), and coefficient of determination (*R*^2^) were calculated. The calculation equations are expressed as follows:

(11) $$MAE=\frac{1}{n}\sum_{i=1}^{n}\left|y_{i}-{\hat{y}}_{i}\right|$$

(12) $$RMSE=\sqrt{\frac{1}{n}\sum_{i=1}^{n}{\left(y_{i}-{\hat{y}}_{i}\right)}^{2}}$$

(13) $$MAPE=\frac{100\%}{n}\sum_{i=1}^{n}\left|\frac{y_{i}-{\hat{y}}_{i}}{y_{i}}\right|$$

(14) $$R^{2}=1-\frac{\sum_{i=1}^{n}{\left(y_{i}-\hat{y}\right)}^{2}}{\sum_{i=1}^{n}{\left(y_{i}-\bar{y}\right)}^{2}}$$

where $y_{i}$ represents the experimental value, ${\hat{y}}_{i}$ represents the simulated value, $\bar{y}$ represents the average experimental value, and n represents the number of data points.

The calculated results showed that the numerical model achieved good prediction accuracy. For compressive strength, the *MAE*, *RMSE*, *MAPE*, and *R*^2^ values were 0.48 MPa, 0.59 MPa, 1.81%, and 0.975, respectively. For splitting tensile strength, the corresponding values were 0.10 MPa, 0.12 MPa, 4.51%, and 0.952, respectively.

These results indicate that the established finite element model can reasonably reproduce the mechanical response of BFRC under different fiber contents and configurations.

Six fiber lengths served as single additions to investigate the influence laws of fiber length on BFRC mechanical properties. These lengths included 6, 9, 12, 15, 18, and 21 mm. The content gradients included 0%, 0.1%, 0.2%, and 0.3%. The hybrid system adopted the combination scheme of 6 mm, 12 mm, and 18 mm determined in the experiment for comparative analysis. Each model group utilized the identical matrix size, material parameters, and boundary conditions. The models changed the fiber length and content parameters only. Table 5 shows the specific details.

### 4.3. Effect of Fiber Parameters on BFRC Strength

#### 4.3.1. Mechanical Properties

**Figure 14:**
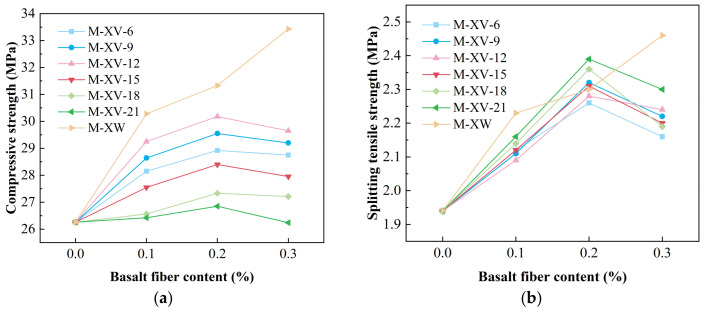
Effect of fiber length on BFRC strength. (**a**) Compression; (**b**) splitting tension.

Figure 14 reflects the variation laws of BFRC mechanical properties under different fiber lengths and contents. Generally, the simulated compressive strength of single-length BFRC showed an initial increase followed by a decrease with the increasing fiber length. Each length group reached a peak value around the 0.2% fiber content. An appropriate fiber amount improved the stress state of the matrix effectively. An excessive content reduced the reinforcement efficiency due to the decline in fiber dispersibility. Fiber length possessed a significant influence on the reinforcement effect. Shorter fibers provided limited constraint on the matrix. Shorter fibers failed to form an effective crack control structure. Longer fibers easily caused entanglement and local agglomeration in the matrix. Medium-length fibers exhibited more stable reinforcement capabilities. Among the numerically investigated single-length fiber cases, the 12 mm fibers exhibited the highest simulated compressive strength. Fibers of different lengths in hybrid BFRC participated in crack suppression and load transfer together. These fibers played a synergistic role at different stages of crack development.

The splitting tensile strength variation law in Figure 14b remained basically identical to the compressive strength. This property showed higher sensitivity to the fiber length. Medium-long fibers provided stronger constraints on crack propagation. These fibers enhanced the tensile properties significantly. The 21 mm fibers showed higher simulated crack-bridging potential due to their larger aspect ratio. The actual construction process faced fiber entanglement and uneven dispersion problems with this length. These issues hindered the uniform molding of concrete. Although the numerical results indicated that 12 mm fibers achieved the highest simulated compressive strength, the experimental program selected 18 mm fibers as the representative single-length fiber, considering practical factors such as fiber availability, dispersion behavior, and construction feasibility.

To mathematically evaluate the mechanism of this “synergistic effect” within the hybrid system, the classical Rule of Mixtures was introduced as a quantitative criterion. Taking the maximum fiber content of 0.3% as a representative baseline, the theoretically expected strength ($f_{the}$) under a non-synergistic, linear additive condition was calculated based on the simulated single-length benchmarks (M-XV3-6, M-XV3-12, and M-XV3-18):

(15) $$f_{the}=0.3\cdot f_{6}+0.4\cdot f_{12}+0.3\cdot f_{18}$$

where $f_{6}$, $f_{12}$, and $f_{18}$ represent the simulated mechanical strengths of concrete reinforced with 0.3% single-length fibers. To quantify the degree of enhancement triggered by the fiber hybridization, the synergy coefficient (α) is defined as the ratio of the composite strength (from numerical simulation) to the theoretically expected value ($f_{the}$). A synergy coefficient (α) greater than 1.0 serves as a strict mathematical indicator of positive synergistic effects, implying that the combination of different fiber lengths yields a mutually beneficial reinforcement action that surpasses a simple linear addition.

For compressive strength at 0.3% dosage, substituting the simulated single-length values (28.75 MPa, 29.65 MPa, and 27.21 MPa, respectively) yields a theoretical linear-addition prediction of 28.65 MPa. Remarkably, the actual experimental strength of Group XW3 (33.00 MPa) and its numerical counterpart M-XW3 (33.43 MPa) exceed this theoretical non-synergistic baseline by 15.2% and 16.7%, respectively. This discrepancy gives a distinct positive compression synergy coefficient.

(16) $$\alpha_{c}=\frac{f_{M-XW,c}}{f_{the,c}}=1.167>1.0$$

where $\alpha_{c}$ denotes the synergy coefficient specifically for the compressive state (where the subscript *c* represents compression) and $f_{M-XW,c}$ represents the numerical compressive strength of the hybrid fiber system.

Similarly, for splitting tensile strength at 0.3% dosage, substituting the corresponding simulated component values (2.16 MPa, 2.24 MPa, and 2.19 MPa) into the linear formula yields a baseline theoretical tensile threshold of 2.20 MPa. Remarkably, the actual experimental splitting tensile strength of Group XW3 (2.23 MPa) and its numerical counterpart M-XW3 (2.46 MPa) exceed this theoretical non-synergistic baseline by 1.36% and 11.8%, respectively. This discrepancy gives a distinct positive tensile synergy coefficient. These clear quantitative gaps mathematically substantiate that the hybrid length configuration triggers a pronounced sequential and complementary reinforcement synergy.

(17) $$\alpha_{t}=\frac{f_{M-XW,t}}{f_{the,t}}=1.118>1.0$$

where the variable $\alpha_{t}$ signifies the synergy coefficient for the splitting tensile state (where the subscript *t* represents tension), and $f_{M-XW,t}$ stands for the numerical splitting tensile strength of the hybrid system.

Therefore, the numerical results mainly provide additional insight into the influence of fiber length, while the experimentally validated conclusions are limited to the investigated fiber lengths. The hybrid system exhibited superior comprehensive performance in both compression and tension during the combination of experimental and numerical simulation results. The hybrid system exhibited improved reinforcement performance compared with single-length fibers.

#### 4.3.2. Analysis of Stress Distribution Characteristics

**Figure 15:**
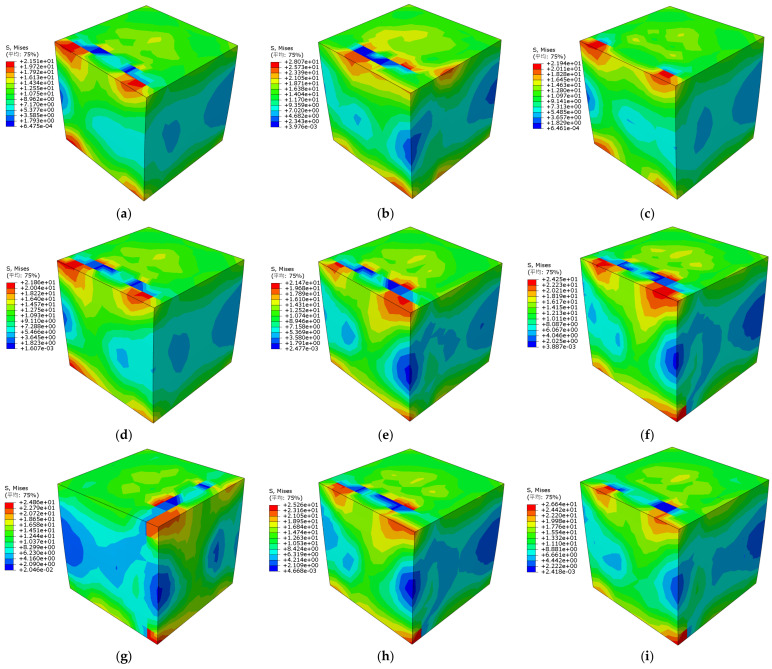
BFRC compressive stress cloud diagram. (**a**) M-XV2-6; (**b**) M-XV2-9; (**c**) M-XV2-12; (**d**) M-XV2-15; (**e**) M-XV2-18; (**f**) M-XV2-21; (**g**) M-XW1; (**h**) M-XW2; (**i**) M-XW3. The Chinese text “平均: 75%” (“Average: 75%”) indicates that the averaging threshold in the ABAQUS post-processing interface was set to 75%.

**Figure 16:**
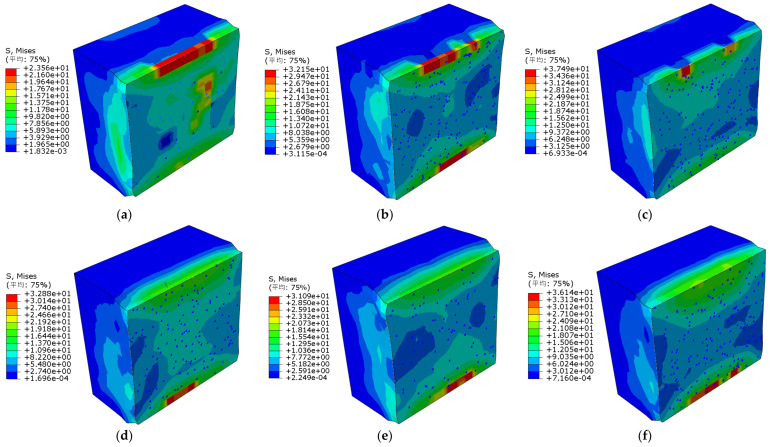

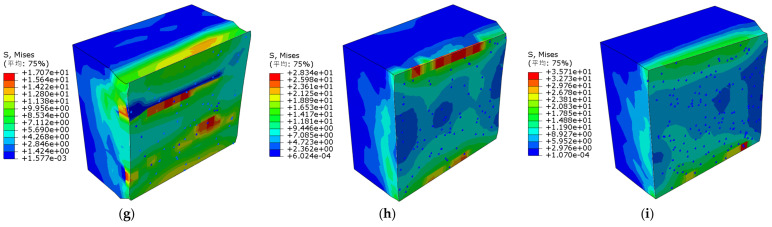
BFRC splitting tensile stress cloud diagrams. (**a**) M-XV2-6; (**b**) M-XV2-9; (**c**) M-XV2-12; (**d**) M-XV2-15; (**e**) M-XV2-18; (**f**) M-XV2-21; (**g**) M-XW1; (**h**) M-XW2; (**i**) M-XW3. The Chinese text “平均: 75%” (“Average: 75%”) indicates that the averaging threshold in the ABAQUS post-processing interface was set to 75%.

Figure 15 and Figure 16 display the von Mises stress contours of BFRC under compressive and splitting tensile loading conditions at peak load. The single-length fiber groups selected the optimal fiber content of 0.2% for stress distribution analysis. To quantitatively evaluate the stress distribution characteristics, the stress fluctuation coefficient ($C_{f}$) was introduced to quantitatively evaluate the distribution uniformity of von Mises stress:

(18) $$C_{f}=\frac{\sigma_{std}}{\sigma_{avg}}$$

where $\sigma_{std}$ and $\sigma_{avg}$ represent the standard deviation and average value of von Mises stress, respectively. A lower $C_{f}$ value indicates a smaller stress fluctuation and a relatively more uniform stress distribution.

Figure 15 illustrates the influence of fiber length and content on stress distribution under compression loading. For the single-length fiber groups (Figure 15a–f), fiber length significantly affected the stress transfer behavior inside BFRC. According to the quantitative results in Table 6, the M-XV2-12 group exhibited a relatively lower $C_{f}$ value compared with other single-length fiber groups, indicating a more uniform stress distribution under compressive loading. The stress contours showed that the high-stress regions were distributed over a relatively wider area, while the local stress concentration was reduced.

For shorter fibers (M-XV2-6), the high-stress regions were mainly concentrated near the loading area, which may be related to the limited crack-bridging effect and stress transfer capacity of shorter fibers. When the fiber length increased to 12 mm, the stress distribution became more continuous, corresponding to the improved compressive strength. However, further increasing the fiber length to 21 mm resulted in increased stress fluctuation, which may be attributed to the reduced dispersion efficiency and local accumulation of long fibers.

For hybrid BFRC, the stress distribution characteristics changed with increasing fiber content (Figure 15g–i). The $C_{f}$ values decreased gradually as the fiber content increased, indicating a reduction in stress fluctuation. The M-XW3 group exhibited the lowest $C_{f}$ value among the hybrid groups, and the stress contours showed a wider distribution of high-stress regions. This result suggests that the hybrid fiber configuration affected the internal stress transfer process and contributed to a more stable stress distribution under compression loading.

Figure 16 presents the von Mises stress distributions of BFRC under splitting tensile loading. The high-stress regions were mainly distributed along the loading strip and crack development areas, which was consistent with the typical splitting failure mode of concrete. The quantitative evaluation results of stress distribution under splitting tensile loading are summarized in Table 7.

For single-length fiber groups, the M-XV2-6 group showed relatively concentrated high-stress regions and a higher stress fluctuation coefficient ($C_{f}$), indicating greater stress fluctuation and local stress concentration. With increasing fiber length from 9 mm to 18 mm, the high-stress regions gradually became more dispersed, and the corresponding $C_{f}$ value decreased. This indicates that appropriate fiber length can improve the stress transfer process and reduce local stress accumulation during splitting failure.

The M-XV2-18 group exhibited a relatively uniform stress distribution under splitting tensile loading, with a lower $C_{f}$ value compared with shorter fibers. This result was consistent with its improved splitting tensile strength. The M-XV2-21 group maintained a comparable stress distribution, although a slight increase in $C_{f}$ was observed, which may be related to the reduced dispersion efficiency of longer fibers in the concrete matrix.

For the hybrid fiber groups, increasing fiber content gradually reduced the concentration of high-stress regions. According to Table 5, the $C_{f}$ value decreased from M-XW1 to M-XW3, and the M-XW3 group exhibited the lowest $C_{f}$ value (0.20). The stress contours showed a more continuous distribution of high-stress regions along the crack propagation path, indicating improved stress transfer behavior. This stress distribution characteristic was consistent with the higher splitting tensile strength of hybrid BFRC. Overall, the numerical results suggest that suitable fiber length combinations and hybrid fiber configurations can reduce stress concentration and may contribute to improved load transfer behavior of BFRC under splitting tensile loading.

### 4.4. Analysis of Synergistic Reinforcement Mechanism in BFRC

#### 4.4.1. Multi-Scale Fiber Synergistic Reinforcement Mechanism

Figure 14 and Figure 15 demonstrate the influence of fiber length on the mechanical properties and internal stress distribution of BFRC. The reinforcement effect of single-length BFRC initially increased and then decreased with increasing fiber length. Medium-length fibers provided a better balance between dispersion and bridging capability. Shorter fibers may be more effective in restricting early crack development, while longer fibers provide stronger crack-bridging ability after crack formation but may reduce dispersion efficiency under certain conditions.

**Figure 17:**
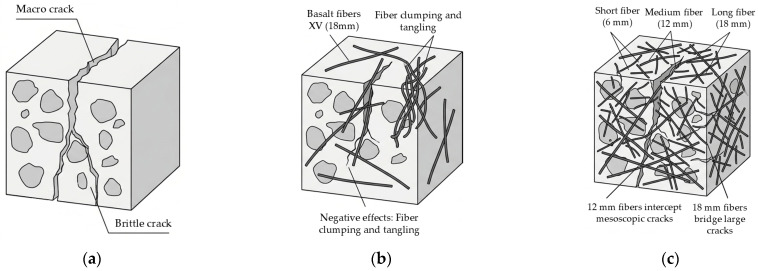
Conceptual illustration of the possible reinforcement mechanism of hybrid BFRC. (**a**) Plain concrete; (**b**) single fiber addition; (**c**) hybrid fibers.

Figure 17 presents a conceptual model illustrating the possible reinforcement mechanism of hybrid BFRC based on the experimental results and numerical analysis. Compared with single-length fibers, the combination of different fiber lengths may contribute to a more stable crack-control process by improving the interaction between fibers and the concrete matrix. The improved mechanical performance of hybrid BFRC may be related to the complementary effects of different fiber lengths during loading.

The stress distribution results suggest that hybrid fibers can contribute to a more uniform stress transfer process and reduce local stress concentration. Therefore, the multi-scale fiber combination provides a possible explanation for the enhanced mechanical behavior of BFRC observed in this study.

#### 4.4.2. Fiber Bridging and Pull-Out Energy Dissipation Mechanism

**Figure 18:**
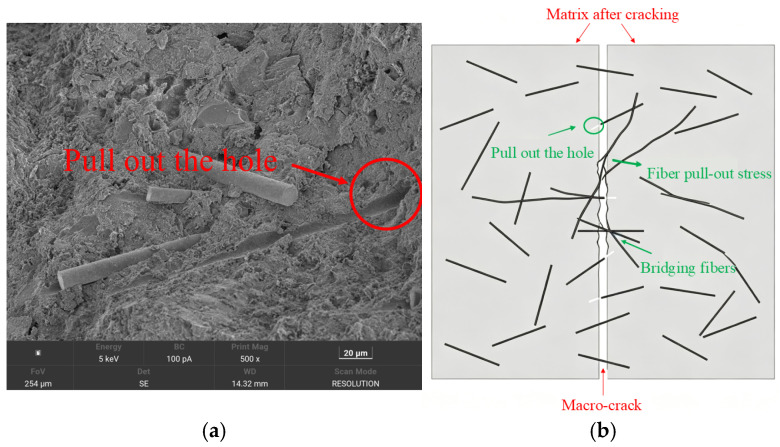
Fiber pull-out and bridging morphology on BFRC fracture surface. (**a**) Microscopic view of fiber pull-out; (**b**) mechanism diagram.

SEM observations on the fracture zone of the XV2 specimens served to reveal the BFRC reinforcement mechanism further. Figure 18 shows the results. Some BFs escaped direct breakage during the crack propagation process. These fibers pulled out from the concrete matrix gradually. This action left distinct pull-out holes on the fracture surface. This phenomenon proved the existence of significant fiber bridging and pull-out energy dissipation during the BFRC failure process [27]. Fibers crossed both sides of the cracks to form a bridging structure when the cracks expanded to the fiber zones. This structure bore part of the tensile stress. The structure limited the crack opening. The fiber–matrix interface suffered gradual debonding with the external load increasing continuously. The fibers began to slip and pull out after this debonding. This displacement process required overcoming the interface bonding force and frictional resistance between the fibers and the matrix. This mechanism consumed a large amount of external input energy [28].

Plain concrete experienced a failure mode of rapid crack coalescing. The fiber bridging action made the crack propagation paths more tortuous than plain concrete. This action extended the crack propagation time. The fibers improved the ductility and toughness of the material effectively. The fiber pull-out process reduced the stress concentration degree at the crack tips significantly. This process allowed more zones to bear the load together. The entire structural stability increased through this mechanism.

In summary, the improved performance of BFRC is the result of the synergistic effect of multi-scale reinforcement mechanisms. At the microscopic level, BF bridging improves the matrix pore structure and enhances interface density. At the mesoscopic level, fiber bridging and pull-out energy dissipation effectively delay crack initiation and propagation. At the macroscopic level, the complementary effects of fibers with different lengths optimizes stress transfer and reduces local stress concentration. Hybrid BFRC outperforms single-doped BFRC in terms of structural uniformity, crack control, and overall stress stability. Therefore, hybrid fibers can achieve pore optimization, crack suppression, and stress redistribution through multi-scale synergistic effects, thereby improving the overall load-bearing capacity and crack resistance of BFRC.

## 5. Conclusions

This study investigated the effects of BF with different contents and length combinations on the mechanical properties of concrete through compressive and splitting tensile tests, SEM microscopic analysis, and numerical simulations. The influence of fiber length combination on the mechanical performance of BFRC was analyzed. The main conclusions include the following points:

(1) Fiber addition improved the mechanical properties of concrete. The compressive and tensile strengths of single 18 mm BFRC showed an initial increase followed by a decrease with the increasing fiber content, and the optimal content was 0.2%. The corresponding strengths reached 26.9 MPa and 2.28 MPa, respectively. The hybrid BFRC exhibited a continuous increase in both compressive and splitting tensile strengths within the fiber content range of 0.1% to 0.3%. At a fiber content of 0.3%, the compressive and splitting tensile strengths reached 33.00 MPa and 2.38 MPa, respectively, indicating improved reinforcement performance compared with the single-length fiber system.
(2) Numerical simulations showed that the compressive strength of single-length BFRC was influenced by fiber length, with an optimal range observed around 12 mm, while the splitting tensile strength increased with increasing fiber length. Compared with single-length fiber systems, the hybrid fiber system showed a more uniform stress distribution and reduced local stress concentration in the numerical results. These findings suggest that the combination of different fiber lengths may contribute to improved stress transfer and crack resistance.
(3) The hybrid BFRC exhibited improved mechanical performance compared with single-length fiber concrete. Based on the Rule of Mixtures, the hybrid system showed a positive synergistic effect, with compression and tensile synergy coefficients reaching 1.167 and 1.118, respectively, indicating that the mechanical performance exceeded the theoretical linear combination of individual fiber systems. The SEM observations and numerical results further suggest that fibers with different lengths may provide complementary reinforcement effects during crack development. However, further quantitative characterization of fiber distribution and orientation is required to better understand the underlying synergistic reinforcement mechanism.

## Figures and Tables

**Table 1:** BFRC mix proportion.

Group	Water	Cement	Sand	Aggregate	Water Reducer	BF-6 mm	BF-12 mm	BF-18 mm
	/kg·m^−3^							
X0	182	330	964	964	3	0	0	0
XV1	182	330	964	964	3	0	0	2.8
XV2	182	330	964	964	3	0	0	5.6
XV3	182	330	964	964	3	0	0	8.4
XW1	182	330	964	964	3	0.84	1.12	0.84
XW2	182	330	964	964	3	1.68	2.24	1.68
XW3	182	330	964	964	3	2.52	3.36	2.52

**Table 2:** Statistical analysis of compressive strength results.

Group	X0	XV1	XV2	XV3	XW1	XW2	XW3
SD/MPa	0.651	0.972	0.865	0.458	0.775	0.682	0.557
$C_{v}$/%	2.52	3.72	3.22	1.71	2.64	2.27	1.69

**Table 3:** Statistical analysis of splitting tensile strength results.

Group	X0	XV1	XV2	XV3	XW1	XW2	XW3
SD/MPa	0.025	0.020	0.031	0.034	0.035	0.025	0.021
$C_{v}$/%	1.34	1.07	1.50	1.79	1.77	1.24	0.94

**Table 4:** Numerical Simulation Parameters.

Concrete Parameter	Value	BF Parameter	Value
Elastic Modulus/GPa	37.96	Elastic Modulus/GPa	76
Poisson’s Ratio	0.2	Poisson’s Ratio	0.25
Dilation Angle/°	30	Filament Diameter/μm	20
Eccentricity	0.1	Tensile Strength/MPa	2500
$f_{b0}/f_{c0}$ Ratio	1.16	Ultimate Elongation/%	3
K	0.667	/	/
Viscosity Parameter	0.0005	/	/

**Table 5:** Numerical simulation scheme.

Model Number		BF Length/mm	BF Content/%
M-X0	M-X0	-	0
M-XV-6	M-XV1-6	6	0.1
	M-XV2-6		0.2
	M-XV3-6		0.3
M-XV-9	M-XV1-9	9	0.1
	M-XV2-9		0.2
	M-XV3-9		0.3
M-XV-12	M-XV1-12	12	0.1
	M-XV2-12		0.2
	M-XV3-12		0.3
M-XV-15	M-XV1-15	15	0.1
	M-XV2-15		0.2
	M-XV3-15		0.3
M-XV-18	M-XV1-18	18	0.1
	M-XV2-18		0.2
	M-XV3-18		0.3
M-XV-21	M-XV1-21	21	0.1
	M-XV2-21		0.2
	M-XV3-21		0.3
M-XW	M-XW1	6, 12, 18	0.1
	M-XW2		0.2
	M-XW3		0.3

**Table 6:** Quantitative evaluation of stress distribution under compression loading.

Model	$\sigma_{avg}$/MPa	$\sigma_{std}$/MPa	$C_{f}$
M-XV2-6	15.8	5.4	0.34
M-XV2-9	16.7	4.9	0.29
M-XV2-12	17.9	3.9	0.22
M-XV2-15	17.1	4.6	0.27
M-XV2-18	16.4	5.0	0.30
M-XV2-21	15.6	5.5	0.35
M-XW1	17.8	4.2	0.24
M-XW2	18.7	3.7	0.20
M-XW3	20.1	3.4	0.17

**Table 7:** Quantitative evaluation of stress distribution under splitting tensile loading.

Model	$\sigma_{avg}$/MPa	$\sigma_{std}$/MPa	$C_{f}$
M-XV2-6	1.62	0.52	0.32
M-XV2-9	1.74	0.45	0.26
M-XV2-12	1.78	0.50	0.28
M-XV2-15	1.81	0.44	0.24
M-XV2-18	1.84	0.43	0.23
M-XV2-21	1.79	0.49	0.27
M-XW1	1.68	0.47	0.28
M-XW2	1.76	0.41	0.23
M-XW3	1.92	0.38	0.20

## Data Availability

The original contributions presented in this study are included in the article. Further inquiries can be directed to the corresponding author.

## Abbreviations

The following abbreviations are used in this manuscript:

BF	Basalt fiber
BFRC	Basalt fiber-reinforced concrete
SEM	Scanning electron microscopy
SD	Standard deviations
ANOVA	Analysis of variance
ITZ	Interfacial transition zone
CDP	Concrete damaged plasticity
C3D8R	Eight-node linear brick elements with reduced integration
T3D2	Two-node linear three-dimensional truss elements
